# Cingulate cortex morphology impacts on neurofunctional activity and behavioral performance in interference tasks

**DOI:** 10.1038/s41598-022-17557-6

**Published:** 2022-08-11

**Authors:** Davide Fedeli, Nicola Del Maschio, Gianpaolo Del Mauro, Federica Defendenti, Simone Sulpizio, Jubin Abutalebi

**Affiliations:** 1 Neuroradiology Department, Fondazione IRCCS Istituto Neurologico Carlo Besta, Milan, Italy; 2 Centre for Neurolinguistics and Psycholinguistics (CNPL), Università Vita-Salute San Raffaele, Via Olgettina, 58, 20132 Milan, Italy; 3 Department of Psychology, University of Milano-Bicocca, Milan, Italy; 4 Milan Center for Neuroscience (NeuroMi), University of Milano-Bicocca, Milan, Italy; 5 The Arctic University of Norway, Tromsø, Norway

**Keywords:** Cognitive neuroscience, Cognitive control

## Abstract

Inhibitory control is the capacity to withhold or suppress a thought or action intentionally. The anterior Midcingulate Cortex (aMCC) participates in response inhibition, a proxy measure of inhibitory control. Recent research suggests that response inhibition is modulated by individual variability in the aMCC sulcal morphology. However, no study has investigated if this phenomenon is associated with neurofunctional differences during a task. In this study, 42 participants performed an Attention Network Task and a Numerical Stroop task in an MRI scanner. We investigated differences in brain activity and response inhibition efficiency between individuals with symmetric and asymmetric aMCC sulcal patterns. The results showed that aMCC morphological variability is partly associated with inhibitory control, and revealed greater activation in individuals with symmetric patterns during the Stroop task. Our findings provide novel insights into the functional correlates of the relationship between aMCC morphology and executive abilities.

## Introduction

Executive Functions (EFs) are a set of versatile control abilities essential for environmental adaptation and self-regulation of cognitive processes^1,2^. A core EF is inhibitory control, that is, the capacity to withhold or suppress a thought or action intentionally. This capacity is often assessed by examining participants’ ability to refrain from producing a prepotent response. Successful inhibition requires participants to maintain awareness of the ongoing performance and consciously suppress inappropriate responses^2–4^. In everyday life, inhibitory control is fundamental for promoting flexible and dynamic adaptations to the environmental demands, which occasionally require one to interrupt automatic but inappropriate behavioral responses (e.g., not crossing the street if a car is passing, even if the pedestrian traffic light is green). Functional neuroimaging research has repeatedly shown that response inhibition engages a network of prefrontal areas including the Anterior Cingulate Cortex (ACC) and the anterior Midcingulate Cortex (aMCC)^5–8^, a large cortical region in the medial wall of the brain^9,10^ (see also^11,12^ for large meta-analyses). The aMCC plays a central role in the detection, monitoring, and mediation of conflicting information during a task. This area is thought to partake in the updating of the current cognitive demands that promotes adaptations and optimizations of goal-directed behaviour^11^. Recently, a growing number of studies have examined the relationship between aMCC and response inhibition by focusing on the impact of the extensive interindividual morphological variability of the aMCC^13–18^. The aim of the present study is to investigate, for the first time, the relationship between the individual variability in the aMCC sulcal pattern and brain functional activity during tasks assessing response inhibition.

The most distinguishable morphological feature of the aMCC is the variable occurrence of the paracingulate sulcus (PCS), a tertiary sulcus that runs dorsal and parallel to the cingulate sulcus (CS) in 30–60% of normal individuals^19,20^. Crucially, the hemispheric distribution of the PCS is determined prenatally and remains stable throughout the life span, being largely unaffected by post-natal brain development and environmental influences^18,21,22^. The PCS is more frequently found in the left (vs. right) hemisphere in the human population^6,19,23^. Based on this finding, sulcal patterns in aMCCs have been traditionally classified into two discrete categories based on the hemispheric distribution of the PCS: (i) PCS asymmetry, when the PCS is present in one hemisphere, but not in the other (i.e., “leftward asymmetry” when the PCS is present only in one left hemisphere, and “rightward asymmetry” when the PCS is present only in one right hemisphere); and (ii) PCS symmetry, when the aMCC sulcal morphology is the same in both hemispheres (i.e., “double absence” when the PCS is absent in both hemispheres, and “double presence” when the PCS is present in both hemispheres)^14,24–26^.

Previous studies have investigated the relationship between the variability of the aMCC sulcal pattern and response inhibition in healthy subjects by using the Stroop^27^ or the Flanker tasks^28–30^. Cachia and colleagues^14^, for example, reported that 5-year-old children with asymmetric aMCC sulcal pattern had a better incongruence score (i.e., incongruent minus congruent trials) during an animal Stroop task, both in terms of lower response times (RTs) and greater accuracy, when compared with same-aged children with a symmetric aMCC sulcal pattern. Similar results were found by adopting the classic color-word Stroop task in 9-year-old children^13,18^ and in young adults^17,18^. In these studies, the aMCC sulcal patterns were reported to explain from 14% up to 27% of the behavioral interference scores variability^13,14,18^, indicating a small but relevant association between individual morphological variability and efficiency in response inhibition. Analogous records of an advantage in inhibitory control associated with asymmetric PCS were also reported for the Flanker task^15,16^. Taken together, these findings suggest behavioral differences in inhibitory control between individuals with symmetric and asymmetric aMCC sulcal patterns, and point towards asymmetry-related cognitive advantages that can be traced back to the early stages of neural development. Besides response inhibition, other EFs such as conflict monitoring and goal maintenance may play a role in explaining differences between conditions in both the ANT and the Stroop tasks. However, according for instance to Miyake and Friedman’s “unity/diversity framework” model^2^, inhibition can be considered a common latent variable shared by all EFs. Therefore, while it is true that interference effects in these tasks can also be ascribed to other EFs or cognitive processes, it seems safe to assume that inhibition is, at least partially, involved in both tasks.

What remains largely unknown is whether the asymmetry-related advantage reported in the literature is backed up by a clear neurofunctional signature, and how variability of the aMCC sulcal pattern impacts the local brain activity while performing tasks involving response inhibition. Should this association be proven significant, it would be the first account of a direct link between prenatally determined aMCC variability, functional activity, and differences in behavioral performance. Individual differences in aMCC sulcal pattern have been previously reported to modulate brain connectivity at rest^31^, as well as the spatial distribution of local task-related clusters of functional activity during decision-making^32^, saccadic and tongue movements^33^, word generation^34^, and pain processing^35^. While these findings are not specific for tasks involving EFs, they represent a promising argument to hypothesize an association between aMCC sulcal pattern and modulation of brain activity during response inhibition. Consistently with the previous literature, in this study we adopted the Attention Network Task ("ANT")^28–30^ to investigate the Flanker effect, and the Numerical Stroop task (also referred to as Counting Stroop task^36,37^) to investigate the Stroop effect during fMRI acquisition. The two tasks entail distinct, although related, dimensions of inhibitory control, respectively “Attention Constraining” (i.e., suppressing interfering information) and “Attention Restraining” (i.e., suppressing automatic responses)^38–41^. During the ANT, participants must answer based on the direction of a central arrow in a string of stimuli, ignoring arrows flanked in the opposite direction. The task requires Attention Constraining since participants have to deliberately constrain their focus to a target element presented among distractors, and suppress interfering information from arrows flanked in the opposite direction during incongruent trials. During the Numerical Stroop task, participants must indicate how many items compose a series of identical numbers or alphabetical characters while ignoring automatic responses based on the number values. The task implies Attention Restraining since participants must refrain from answering with prepotent (but inappropriate) automatic responses in favor of novel, goal-directed responses.

In the present study, reaction times and neurofunctional activity will be compared between individuals with symmetric and asymmetric aMCC sulcal patterns. In line with previous findings, we expect greater efficiency in inhibitory control (i.e., faster RTs and better accuracy) for individuals with asymmetric sulcal profiles compared to those with symmetric profiles. Similarly, we expect individuals with asymmetric profiles to show more pronounced evidence of efficient response inhibition when plotting differences between conditions as a function of response time (i.e., delta plotting). As for functional activations, increased aMCC and pre-supplementary motor area activity has typically been associated with greater cognitive load and task difficulty^9,42,43^. Therefore, we expect greater functional activity in these regions associated with poorer inhibitory control in individuals with symmetric sulcal patterns. Finally, brain-behavior correlations will be performed to further test the relationship between aMCC functional activity and behavioural responses.

## Materials and methods

### Participants

Forty-three Italian young adult participants were recruited. One subject was excluded from the analyses due to white matter hyperintensities, thus resulting in a final sample of 42 participants (mean age: 25.19 ± 4.89; 30 F). All participants had no history of neurological or psychiatric disorders, had normal or corrected-to-normal vision, and were right-handed^44^. For each participant the following measures were obtained: Socio-Economic Status (SES) (The MacArthur Scale of Subjective Social Status, https://macses.ucsf.edu/research/psychosocial/subjective.php#measurement) (mean years of formal education: 16.62 ± 1.45; mean personal income score: 1.52 ± 0.77; mean family income score: 3.62 ± 1.08); Fluid intelligence quotient (Raven’s Standard Progressive Matrices for adults^45^) (mean corrected score: 33.54 ± 2.52); visuo-spatial working memory (Corsi test^46^) (mean Corsi forward corrected score: 6.27 ± 1.16; mean Corsi backward corrected score: 5.27 ± 0.86). No participant was discarded because of low intelligence quotient or low working memory score.

The study was carried out in accordance with the Declaration of Helsinki and with the ethical approval from the Human Research Ethics Committee of the Vita-Salute San Raffaele University, Milan, Italy. All participants gave written informed consent.

### Procedure

Participants performed two tasks inside of an MRI scanner. The order of the tasks was counterbalanced across participants, and the two tasks were separated from each other by a T1 structural sequence of the duration of 7.83 min. The study was performed with Presentation software (https://www.neurobs.com, version 20.3, build 02.25.2019).

The Attention Network Task^29,30^ expands the classic Flanker task^28^ and allows one to investigate the involvement of three different attentional networks/functions: alerting, orienting, and executive control. Attention alerting represents the ability of reaching and maintaining an alerted state; attention orienting represents the ability of selecting specific information from an input; executive control represents the ability of solving conflict selecting only the appropriate responses. As we were mainly focused on investigating the interaction between the aMCC sulcal pattern and the functional activity associated with inhibitory control (i.e., executive control), other effects associated with visual priming cues in the ANT (i.e., alerting and orienting effects) were not considered here and reported in supplementary materials^15,16^. The Attention Network Task (ANT) was adapted from Abutalebi et al.^47^. Two 7 m 43 s runs, each comprising 96 trials, were presented. The two runs were separated by a small break of 30 s, or more if response box position adjustment was required. The experiment was preceded by a short practice session of 16 trials. In each trial participants were shown a sequence of five arrows aligned horizontally and were instructed to answer as fast and accurately as possible based on the direction of the central arrow by pressing the left or right button of a response box. Stimuli were presented in congruent, incongruent, or neutral conditions (64 trials per each condition, pseudorandomized order). Congruent trials consisted in a sequence of arrows all flanked in the same direction (→ → → → →), incongruent trials consisted in a sequence of arrows with the central arrow flanked in opposite direction with respect to the central arrow (← ← → ← ←), and neutral trials consisted in a sequence of lines with only the central arrow flanked in one direction (– – → – –). For each condition, target stimuli were presented in 50% of the cases above a central fixation cross (up) and in the other 50% below the central fixation cross (down). Stimuli were preceded by a fixation cross ( +) (duration = 400 ms) at the center of the screen, and a visual cue (duration = 100 ms). Four visual cue conditions were adopted: no cue, center cue, double cue, and spatial cue. In the no cue condition, participants saw only the fixation cross for 100 ms after its original 400 ms presentation. In the center cue condition, an asterisk (*) was presented at the center of the screen, in place of the fixation cross for 100 ms. The double cue condition had identical timing, but participants saw two asterisks (*) above and below a central fixation cross, in the position corresponding to the two possible target stimuli locations. In the spatial-cue condition, an asterisk was presented above or belove the central fixation cross for 100 ms, anticipating the target position (see Figure S1 in supplementary materials). The spatial cues were always valid (i.e., correctly anticipated the target stimulus position). Target stimuli lasted for 1700 ms and remained displayed on the screen after the participant’s response until the end of the presentation time. Inter stimulus interval (ISI) corresponded of a black screen and was jittered with Dale’s exponential function^48^ (mean ISI = 2797.66 ms; min ISI = 1873; max ISI = 4964 ms). RTs and accuracy scores were recorded for each trial.

The Numerical Stroop task^27,37^ was adapted from Hernández et al.^49^. Participants were presented two 7 m 48 s runs. The two runs were separated by a small break of 30 s, or more if response box position adjustment was required. Each run consisted of 108 trials, and the experiment was preceded by a short practice session of 16 trials. In each trial, participants were asked to indicate the number of items composing a series of one, two, three, or four identical numbers (or alphabetical characters), by using the first, second, third, or fourth button of a response box. Stimuli were presented in congruent, incongruent, or neutral conditions (72 trials per condition, pseudorandomized order, stimulus duration = 2000 ms). During congruent trials, the number of items corresponded to the number values (i.e., 1; 22; 333; 4444); during incongruent trials, the number of digits was different from the number values (e.g., 11; 2222; 3; 444); during neutral trials, alphabetical characters were presented (e.g., Z; GG; MMM; ZZZZ). Stimuli were preceded by a central fixation cross (duration = 500 ms). Stimuli remained displayed on the screen after participant’s response until the end of the presentation time. RTs and accuracy scores were recorded for each trial. Stimuli were followed by a jittered ISI^48^ (mean ISI = 1770.11 ms; min ISI = 1036 ms; max ISI = 4113 ms) consisting of a black screen.

### MRI acquisition

MRI acquisition was performed at the Centro di Eccellenza Risonanza Magnetica ad Alto Campo (C.E.R.M.A.C., Unit of Neuroradiology) San Raffaele Hospital, Milan (Italy) with a 3 Tesla Philips Ingenia CX MR scanner (Philips Medical Systems, Best, Netherlands) with a 32 channels SENSE head coil.

For both the ANT and the Numerical Stroop tasks, functional scans were acquired with a fast speed Echo Planar Imaging (EPI) sequence (Echo Time [TE] = 33 ms; Repetition Time [TR] = 2000 ms; Flip Angle [FA] = 85°; number of volumes per run = 236 (ANT); 256 (Numerical Stroop); Field of View [FOV] = 240 × 240; matrix size = 80 × 80; 35 axial slices per volume; slice thickness = 3; interslice gap = 0.75; voxel size = 3 × 3 × 3; Phase Encoding direction [PE] = A/P; SENSE factor = 2; whole brain coverage). Five dummy scans preceded each run to optimize EPI image signal.

A high-resolution Magnetization Prepared Rapid Gradient Echo (MPRAGE) T1-weighted anatomical image was acquired for each participant with the following parameters: repetition time (TR) = 9.9 ms, echo time (TE) = 4.9 ms, flip angle = 8°, FOV = 269 mm, matrix size = 384 × 384, number of axial slices = 243, slice thickness = 1.4 mm, voxel size = 0.7 × 0.7 × 0.7 mm 3, Phase Encoding direction (PE) = A/P, SENSE factor = 2, with whole brain coverage.

### ACC sulcal pattern classification

For all T1-weighted structural images the origin was set to match the bicommissural line (anterior commissure-posterior commissure). Sulcal pattern classification was then performed following Garrison’s protocol^50^. Images were imported into MANGO (Multi-image Analysis GUI, v 4.0, http://ric.uthscsa.edu/mango/mango.html) and the PCS was identified as the sulcus running dorsal and parallel to the cingulate sulcus for most of its length. The anterior limit of the PCS was identified on the sagittal plane at x = − 5 mm for the left hemisphere, and x = + 5 mm for the right hemisphere, starting from the point at which the sulcus begins to move on a rostro-caudal direction from the imaginary extension of the bicommissural line. The posterior limit of the PCS was identified as a line passing through the anterior commissure and perpendicular to the bicommissural line. The PCS was then measured and classified as ‘‘present’’ (PCS ≥ 20 mm) or ‘‘absent’’ (PCS < 20 mm). When the PCS was interrupted, sulcal sections were considered only if interruptions were ≤ 19 mm. Interruptions were not included in the computation of the total length of the PCS. Participants were classified as “asymmetric” when the PCS was present in only one hemisphere, but not in the other, and “symmetric” when the PCS was bilaterally present or bilaterally absent.

### fMRI pre-processing

Functional data for both the ANT and Numerical Stroop tasks were processed by adopting the surface-based fMRI pipeline developed by Brodohel and colleagues^51^. With respect to standard volumetric processing, surface-based fMRI is supposed to greatly increase the anatomical precision of the functional findings. As a matter of fact, spatially smoothing volumetric data increases the risk of signal contamination^52^ between anatomically distant regions. This is particularly true for functional regions that may be adjacent in the folded cortex (i.e., volumetric space) but are separated in the unfolded cortex (i.e. surface space), such as the cingulate and paracingulate gyri when a PCS is present in the same hemisphere. Therefore, this approach better accounts for the individual variability in gyrosulcal morphology and allows to disentangle the specific contribution of neighboring functional regions on the aMCC. Moreover, since the left and right aMCC are very close to each other in the volumetric space, bilateral aMCC activation patterns are often the consequence of the relatively large smoothing kernel (e.g., 8 × 8 × 8 mm^3^ or 6 × 6 × 6 mm^3^) that is adopted in classic volume-based analyses. While necessary for improving the signal-to-noise ratio, this methodological preprocessing step largely increases the chances of erroneously spreading functional activity located on one hemisphere onto the contralateral cortex. Surface-based fMRI combined with a small smoothing kernel (3 × 3 × 3 mm^3^) largely prevents this type of inter-hemispheric signal contamination. The following processing steps were performed: individual surface estimation; slice timing correction of functional data; spatial realignment and coregistration to skull-stripped bias corrected T1-weighted structural image; General Linear Model (GLM) estimation; mapping of the functional contrast-images in the native volumetric space to the individual surface; normalization and smoothing. T1-weighted structural images were segmented with the Computational Anatomy Toolbox (CAT12 v1429, http://www.neuro.uni-jena.de/cat/) based on SPM12 v7219 (www.fil.ion.ucl.ac.uk/spm/). Structural images were segmented into gray matter (GM), white matter (WM) and cerebrospinal fluid (CSF), resulting in separate single-subject image volumes for each tissue class. CAT12 segmentation approach uses a spatial adaptive non-local mean (SANLM) denoising filter and a local adaptive segmentation (LAS) that applies a local intensity transformation of tissue classes to correct for regional inhomogeneities and intensity variations. Additionally, an Adaptive Maximum A Posterior (AMAP) technique^53^ and a Partial Volume Estimation (PVE)^54^ were carried out in order to obtain a more accurate segmentation. Central surface reconstruction was carried out for both hemispheres of each structural image using the CAT12 standard pipeline that employs a projection-based thickness (PBT) computation approach^55^. A central surface mesh representing the distance between the inner (GM/WM) and outer (GM/CSF) boundaries of the cortex was generated for each participant. This approach allows to work with vertices, instead of voxels, whose computation is unbiased by potential partial volume effects detectable in blurred sulcal regions. Functional images were slice-time corrected and realigned to the first volume and unwarped to correct for motion artifacts and geometric distortions. Realigned functional volumes were coregistered to the bias-corrected structural brain image. Functional images were then entered in a separated GLM for each task. BOLD signal was convolved using the Canonical Hemodynamic Response Function (HRF), and a 128 s high-pass filter was applied to the timeseries. Serial correlations were accounted for using the AR (1) model during parameter estimation. For each task, onsets for the Congruent, Incongruent, and Neutral conditions were entered into the model. Realignment parameters for the two sessions were entered as nuisance covariates. The following directional t-contrasts were estimated: incongruent > congruent; incongruent > neutral; congruent > neutral. Contrast-images estimated at the first level were then mapped to each participant’s individual surfaces generated with CAT12, with absolute maximum option. Surface images were than resampled to the standard 32 k Human Connectome Project template provided by CAT12, and smoothed with a 3 mm × 3 mm × 3 mm Full-Width at Half-Maximum (FWHM) gaussian kernel, as recommended by Brodohel and colleagues^51^.

Despite the great advantages of performing surface-based rather than volume-based fMRI analyses, the latter approach is still the most adopted and is considered a gold standard. As a control analysis, a volumetric processing pipeline was also implemented with standard settings: slice-time correction; realignment and unwarping; segmentation of the structural image; coregistration to the reference bias-corrected skull-stripped structural image; normalization to the standard MNI volumetric template; and smoothing with a 8 mm × 8 mm × 8 mm FWHM gaussian kernel. Results from this control pipeline are reported in supplementary materials.

### Statistical analyses

#### Behavioral analyses

For both the ANT and the Numerical Stroop task, statistical analyses were run to test the effects of aMCC sulcal pattern on the executive performance. Since average accuracy was very high (i.e., reaching “ceiling effect”) in both tasks (ANT mean accuracy score = 99.65%; Numerical Stroop mean accuracy score = 99.10%), only RTs were considered for the analyses.

We ran a set of general linear mixed-effects models separately for each task. All analyses were performed using R (R Core Team 2015). The models were fitted using the lmer function implemented in the lme4 package (version 1.1–27^55–57^).

For each task, a linear mixed-effects model was run using participants’ RTs to correct responses as a dependent variable, with task condition (“congruent” vs. “incongruent” vs. “neutral”; reference level = congruent), aMCC sulcal pattern (“PCS asymmetry” vs. “PCS symmetry”; reference level = “PCS asymmetry”), and their two-way interaction as predictors. Likelihood ratio tests were performed to assess the significance of the fixed effects. For each fixed term we compared models in which that term was present versus absent. Fixed terms were retained only when their exclusion would significantly diminish the goodness of fit. In case of significant interactions, all lower-order terms were maintained in the final model. The model also included by-participants random intercepts. Coefficients were considered as significant when *t* ≥|2|. In addition, we sorted RTs by speed from the fastest to the slowest in bins of equal size. We included these quantiles in linear mixed-effect models to evaluate how distributions differ as a function of response speed. Quantile analyses are informative about whether the difference between task conditions is significantly larger for specific latencies only (e.g., the slowest responses), or such an effect is visible across all responses. Moreover, we investigated differences in the distribution of RTs between conditions by means of delta plots^56–58^. Delta plotting is an increasingly used graphical and inferential method that allows one to describe temporal dynamics of cognitive processing by plotting task effects as a function of response time (i.e., quantiles)^58^. By visually comparing the slopes of incongruency effects against groups and with known literature-based trends, delta plots can be highly informative in revealing differences in the modalities and the extent of inhibitory control processes taking place during the task. Incongruency effects associated with Stroop and Flanker effects usually have a growing slope, indicating increasing spread between conditions as response latency grows^58–62^. Conversely, the reduction of the incongruency effect typically observed in the latest quantiles of interference tasks (a decrement or a reversal in the separation between conditions for the slowest RTs) is thought to reflect the use of a slow but effective inhibition suppression mechanism^58,60,61^. Since delta plots allow to analyze the intervention of control/suppression mechanisms, in the present study, we used them to investigate whether people with different ACC morphologies differ in how they implement these mechanisms. RTs of correct responses in each condition for each participant were sorted from the fastest to the slowest and grouped in five equal-sized bins. The 1st quantile would consist of the fastest 20% of the responses from a given participant in a specific condition; the 2nd quantile the next fastest 20%, and so on, until the 5th quantile, which would consist of the slowest 20% of the responses. Quantiles were then included in a second linear mixed-effects model as a fixed effect. Nonlinear relationships were tested by comparing this model with one in which nonlinearities were fitted by using orthogonal quadratic polynomials for the quantile fixed effect. Delta plots representing the difference between conditions as a function of RTs quantiles were generated for each task and separately for individuals with symmetric and asymmetric aMCC sulcal patterns.

#### Neuroimaging analyses

At the second-level, smoothed and resampled individual con-images were entered into a GLM and a set of one-sample t-test were performed in order to test the effects of task conditions irrespective of aMCC sulcal pattern. Subsequently, a full factorial design was used to investigate differences in brain activity between individuals with symmetric and asymmetric aMCC sulcal patterns. As we were interested in studying the impact of local morphological variability on the functional activity of the cingulate/prefrontal cortex, group analyses were performed within an inclusive mask created mapping to the surface the following bilateral regions from the Harvard–Oxford atlas (distributed with the FMRIB Software Library FSL^63^): Cingulate Gyrus, anterior division; Paracingulate Gyrus; Superior Frontal Gyrus; Frontal Pole. Atlas-to-surface mapping was performed with CAT12, using the standard 32 k Human Connectome Project template as a reference. For the group analysis gender and years of formal education were entered as nuisance covariates. Gender was considered as a covariate because of evidence of gender-related differences in aMCC sulcal pattern distribution (see^20,64,65^) and cortical complexity^66–68^. Years of formal education were entered in the model as a covariate, since education has been reported to impact on Stroop RTs^69^. Age was not entered as a covariate to avoid multicollinearity since it was highly correlated with years of formal education (R = 0.61, p < 0.001). For all the analyses the statistical threshold was set at p < 0.05 family-wise error (FWE) corrected for multiple comparisons at the cluster level, and at p < 0.001 uncorrected at the vertex level (i.e., statistically equivalent to uncorrected at the voxel level for volume-based analyses).

#### Brain-behavior interactions

A correlation analysis was performed to test whether the individual mean functional activity from clusters resulting from the group contrasts was correlated with the mean RTs associated with that effect in the two tasks. The same correlation analysis was also performed considering mean RTs from the slowest quantiles (4th and 5th), since the leveling-off of the slope for slow responses is typically associated with the efficiency of response inhibition^58,61,70^.

## Results

### aMCC sulcal pattern classification

Asymmetric aMCC sulcal patterns were reported for 20 participants (47.62%), while symmetric patterns were reported for 22 participants (52.38%). For the asymmetric pattern, leftward asymmetry was observed in 12 participants (28.57%), and rightward asymmetry in 8 participants (19.05%). For the symmetric pattern, a double PCS presence was observed in 8 participants (19.05%), and a double PCS absence in 14 participants (33.33%). A chi-square (χ^2^) analysis revealed that sulcal patterns were equally distributed when considering asymmetry (i.e., asymmetry, symmetry; χ^2^(1) = 0.09, *p* = 0.76). No significant difference in age, gender, handedness, education, SES, fluid intelligence quotient, and visuo-spatial working memory was found between the two groups (all χ^2^ s < 1; all *p*s > 0.3; all *t*s < 1).

### Behavioral analyses

#### ANT

**Figure 1:**
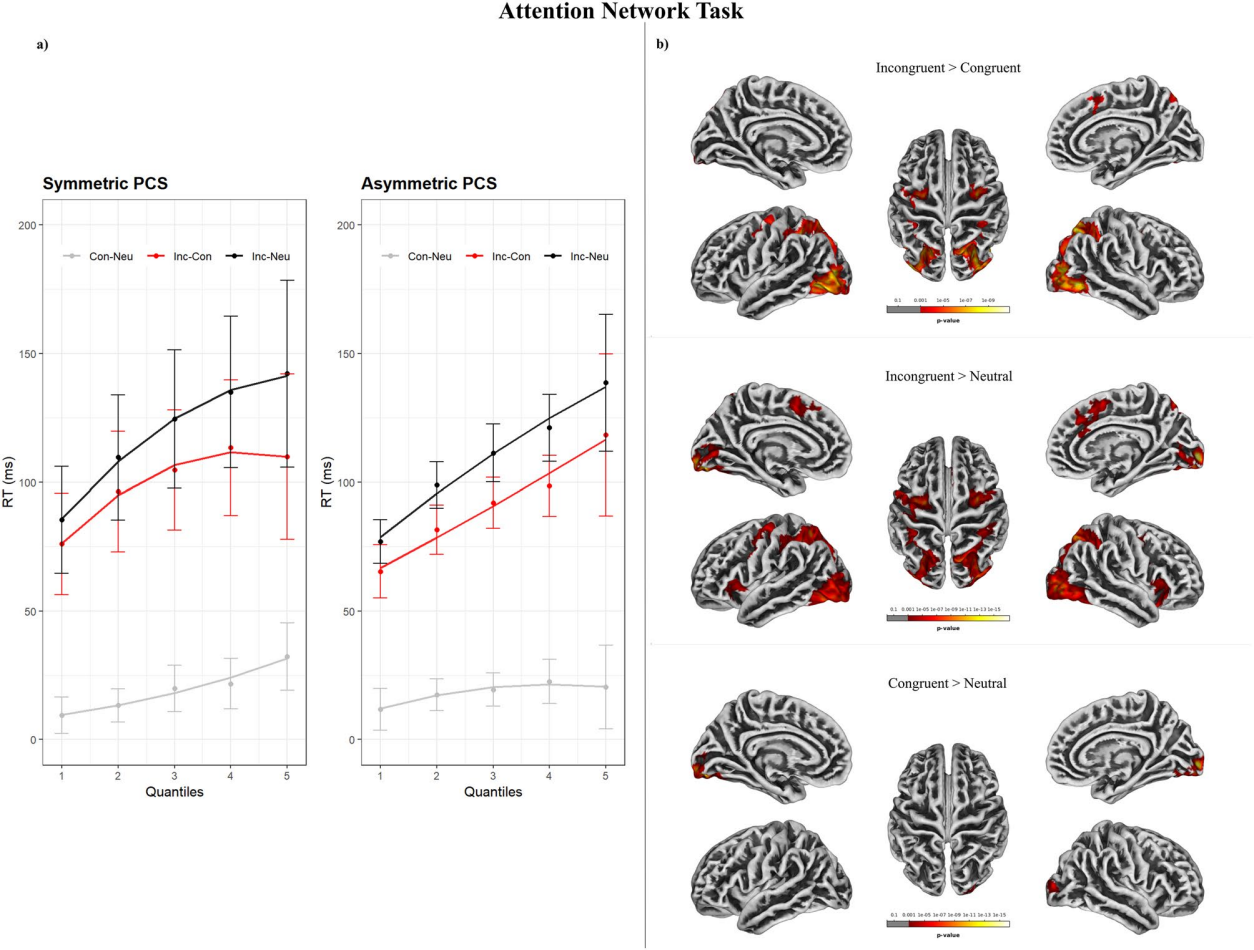
(**a**) Delta plots of the effects detected in the Attentional Network Task (ANT), as a function of quantiles. (**b**) Brain activity during the ANT. Significant results are shown at cluster level FWE-corrected for multiple comparisons p-value < 0.05, and vertex level uncorrected p-value < 0.001. *Inc* Incongruent, *Con* Congruent, *Neu* Neutral.

 The model to test for the effect of ACC sulcal pattern asymmetry on mean RTs in the ANT showed a significant effect of Condition (χ^2^ (2) = 1657.79, p < 0.001), with faster responses for neutral trials (M = 552 ms, SD = 126) compared to congruent trials (M = 571 ms, SD = 129, *b* = − 18.84, SE = 2.86, *t* = − 6.59) and faster responses for congruent trials than incongruent trials (M = 667 ms, SD = 150, *b* = − 95.75, SE = 2.86, *t* = − 33.45). No effect of aMCC sulcal asymmetry was found (χ^2^ (1) = 0.92, p = 0.34), nor a 2-way interaction (χ^2^ (1) = 3.2, p = 0.2). The model including RTs quantiles as a fixed factor revealed a significant Condition × Quantile 2-way interaction (χ^2^ (2) = 150.25, p < 0.001); and a significant aMCC sulcal asymmetry × Condition × Quantile 3-way interaction (χ^2^ (4) = 41.81, p < 0.001). Including the second-order polynomial in fitting RTs quantiles significantly increased the goodness of fit of the model (χ^2^ (6) = 295.47, p < 0.001). Delta plots are reported in Fig. 1; Conditions-by-quantile plots are reported in Figure S2 in supplementary materials.

Delta plots revealed that the incongruent > congruent (incongruency) effect increased linearly in individuals with asymmetric patterns. The incongruency effect increased nonlinearly in individuals with symmetric patterns, with a flatter slope that decreased in the fifth quantile associated with the slowest responses. The incongruent > neutral effect increased linearly in individuals with asymmetric patterns. Individuals with symmetric patterns also showed a positive effect growth, with a nonlinear slope that flattened in the latest quantile. The congruent > neutral effect was constant in individuals with asymmetric patterns with an almost flat slope across all quantiles. The same effect increased linearly across quantiles in individuals with symmetric patterns.

#### Numerical stroop

**Figure 2:**
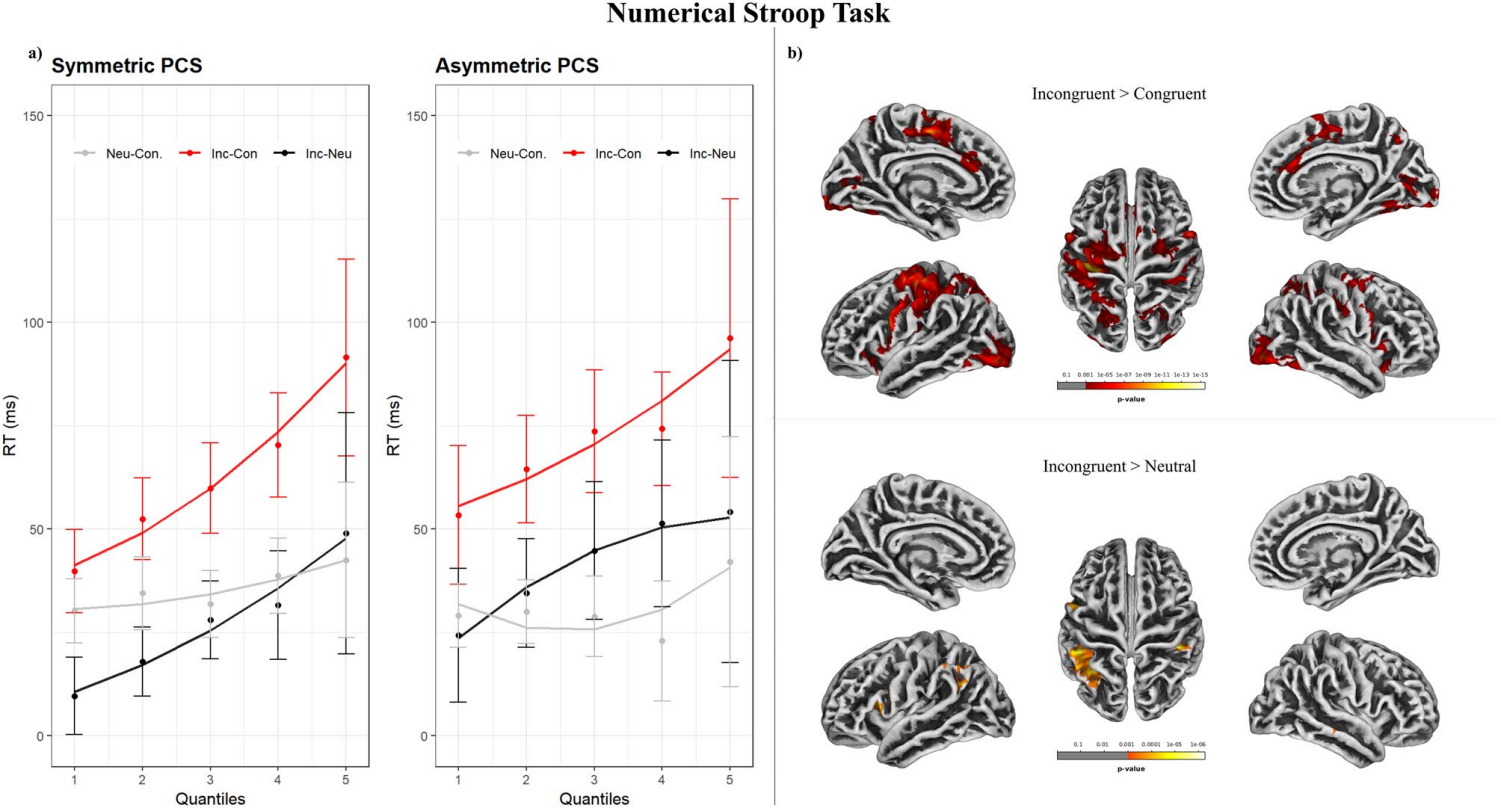
(**a**) Delta plots of the effects detected in the Numerical Stroop task, as a function of quantiles. (**b**) Brain activity during the Numerical Stroop task. *Inc* Incongruent, *Con* Congruent, *Neu* Neutral.

The model to test for the effect of aMCC sulcal pattern asymmetry on RTs in the Numerical Stroop task showed a main effect of Condition (χ^2^ (2) = 348.12, p < 0.001), with faster responses for congruent trials (M = 639 ms, SD = 155) compared to neutral trials (M = 673 ms, SD = 164;*b* = − 33.59, SE = 3.54, *t* = − 9.5) and faster responses for congruent trials than incongruent trials (M = 707 ms, SD = 172; *b* = − 66.91, SE = 3.55, *t* = − 18.84). No significant effect of aMCC sulcal asymmetry (χ^2^ (1) = 2, p = 0.16) nor a 2-way interaction (χ^2^ (2) = 4.69, p = 0.1) were found. The model including RTs quantiles as a fixed factor revealed a significant Condition x Quantile 2-way interaction (χ^2^ (2) = 63.81, p < 0.001), and a significant aMCC sulcal asymmetry x Condition x Quantile 3-way interaction (χ^2^ (4) = 28.48, p < 0.001). Including the second-order polynomial in fitting RTs quantiles significantly increased the goodness of fit of the model (χ^2^ (6) = 691.33, p < 0.001). Delta plots are reported in Fig. 2. The incongruent > congruent effect (i.e. “incongruency” effect) increased across quantiles for both individuals with asymmetric and symmetric patterns with a similar linear slope. The incongruent > neutral effect (i.e. “interference” effect) increased nonlinearly in individuals with asymmetric patterns, flattening from the third quantile onwards. The effect increased linearly across quantiles in individuals with symmetric patterns. The neutral > congruent effect was stable for both individuals with asymmetric and symmetric patterns.

### Neuroimaging analyses

#### ANT

The incongruent > congruent contrast, irrespective of PCS asymmetry, resulted in the activation of fronto-occipital regions, including the right paracingulate gyrus and frontal orbital cortex. A similar fronto-occipital pattern was found for the incongruent > neutral contrast, with increased activity in the bilateral paracingulate gyrus, superior frontal gyrus, frontal orbital cortex and supplementary motor area (juxtapositional lobule cortex). The congruent > neutral contrast revealed the activation of posterior occipital regions and of the left superior parietal lobule. When inspecting differences in brain activity in individuals with symmetric and asymmetric PCS profiles, no significant effect was found. Results are reported in Table 1 and Fig. 1

**Table 1:** t-contrast results for the effects detected in the ANT task.

ANT										
Contrast	Hemisphere	Region (Harvard–Oxford)	Cluster p (FWE-corr)	k (mm^3^)	T value	Z score	Peak p (unc)	x	y	z
Congruent > Neutral	L	Occipital Fusiform Gyrus	< 0.001	439	12.26	Inf	< 0.001	− 19	− 83	− 13
	R	Occipital Fusiform Gyrus	< 0.001	279	11.7	7.71	< 0.001	23	− 79	− 12
	R	Occipital Pole	< 0.001	94	11.62	7.68	< 0.001	11	− 96	− 7
	R	Occipital Pole	< 0.001	90	5.63	4.81	< 0.001	30	− 93	8
	L	Superior Parietal Lobule	0.025	30	4.84	4.28	< 0.001	− 27	− 54	46
	R	Intracalcarine Cortex	0.15	21	4.57	4.09	< 0.001	15	− 78	11
	L	Lateral Occipital Cortex	0.013	33	4.55	4.07	< 0.001	− 26	− 83	17
	L	Intracalcarine Cortex	< 0.001	50	4.16	3.78	< 0.001	− 6	− 87	3
Incongruent > Neutral	R	Occipital Pole	< 0.001	238	13.03	Inf	< 0.001	11	− 96	− 5
	L	Occipital Fusiform Gyrus	< 0.001	3019	12.41	Inf	< 0.001	− 21	− 82	− 18
	R	Occipital Fusiform Gyrus	< 0.001	2762	9.74	6.97	< 0.001	24	− 78	− 14
	R	Middle Frontal Gyrus	< 0.001	400	8.76	6.54	< 0.001	33	− 2	50
	L	Superior Frontal Gyrus	< 0.001	849	8.3	6.32	< 0.001	− 22	− 4	49
	L	Frontal Orbital Cortex	< 0.001	249	6.75	5.51	< 0.001	− 31	26	− 6
	R	Paracingulate Gyrus	< 0.001	359	6.47	5.34	< 0.001	5	17	43
	R	Frontal Orbital Cortex	< 0.001	534	6.24	5.2	< 0.001	26	14	− 19
	L	Paracingulate Gyrus	0.048	27	6.07	5.1	< 0.001	− 10	45	12
	L	Juxtapositional Lobule Cortex	< 0.001	163	5.7	4.86	< 0.001	− 5	5	45
	R	Precentral Gyrus	< 0.001	107	5.44	4.69	< 0.001	47	9	29
	L	Juxtapositional Lobule Cortex	< 0.001	50	5.14	4.49	< 0.001	− 6	− 7	49
	R	Superior Frontal Gyrus	0.012	34	4.49	4.03	< 0.001	13	10	66
	R	Intracalcarine Cortex	0.01	35	4.32	3.9	< 0.001	15	− 61	6
Incongruent > Congruent	R	Lateral Occipital Cortex, inferior division	< 0.001	2151	8.3	6.32	< 0.001	52	− 68	− 8
	L	Inferior Temporal Gyrus, temporooccipital part	< 0.001	2006	8.26	6.3	< 0.001	− 42	− 62	− 3
	R	Precentral Gyrus	< 0.001	233	6.89	5.58	< 0.001	32	− 4	46
	L	Precentral Gyrus	< 0.001	279	5.55	4.76	< 0.001	− 24	− 5	49
	L	Postcentral Gyrus	0.004	39	5.27	4.58	< 0.001	− 46	− 28	35
	L	Precentral Gyrus	0.019	31	4.67	4.16	< 0.001	− 57	6	32
	R	Paracingulate Gyrus	< 0.001	82	4.27	3.86	< 0.001	6	23	47
	R	Frontal Orbital Cortex	0.008	35	4.24	3.84	< 0.001	31	25	− 7
	R	Precentral Gyrus	0.002	43	4.03	3.67	< 0.001	46	8	35

Significance threshold is set at vertex-p-uncorrected < 0.001 and cluster-p-FWE-corrected < 0.05. Only one local maximum per significant cluster is listed.

*R* Right hemisphere, *L* Left hemisphere.

#### Numerical stroop

**Figure 3:**
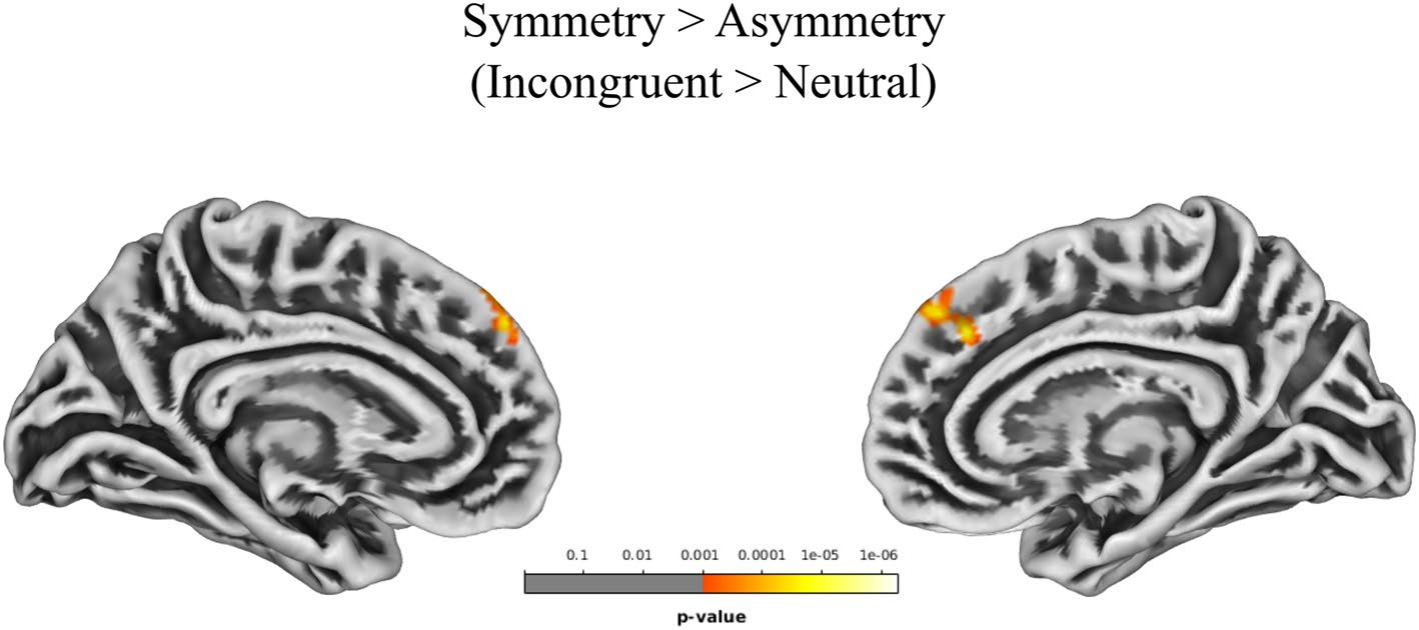
Difference in brain activity between individuals with Symmetric and Asymmetric aMCC sulcation patterns for the Incongruent > Neutral contrast. Significant results are shown at cluster level FWE-corrected for multiple comparisons p-value < 0.05, and vertex level uncorrected p-value < 0.001.

The incongruent > congruent (incongruency) contrast, irrespective of PCS asymmetry, resulted in the activation of frontal, insular, parietal, and occipital cortices, including the left paracingulate gyrus, the bilateral anterior cingulate and frontal orbital cortices, and the right superior frontal gyrus and supplementary motor area. The incongruent > neutral (interference) contrast resulted in the activation of frontal, parietal and occipital areas, including the left inferior frontal gyrus, but not medial aspects of the frontal cortex. The congruent > neutral contrast did not reveal any significant result. When investigating differences in brain activity between individuals with symmetric and asymmetric PCS profiles, a significant difference emerged for the incongruent > neutral contrast. Individuals with symmetric profiles showed greater activity in the right paracingulate cortex and in the left medial part of the superior frontal gyrus with respect to individuals with asymmetric profiles. Results are reported in Table 2, Figs. 2 and 3; see also Figure S3.

**Table 2:** t-contrast results for the effects detected in the Numerical Stroop task.

Contrast	Hemisphere	Region (Harvard–Oxford)	Cluster p (FWE-corr)	k (mm^3^)	T value	Z score	Peak p (unc)	x	y	z
**Stroop**										
Congruent > Neutral	–	–	–	–	–	–	–	–	–	–
Incongruent > Neutral	L	Inferior Frontal Gyrus, pars opercularis	< 0.001	67	5.33	4.62	< 0.001	− 45	16	9
	R	Postcentral Gyrus	< 0.001	91	5.31	4.6	< 0.001	46	− 30	46
	L	Supramarginal Gyrus, anterior division	< 0.001	691	5.21	4.54	< 0.001	− 52	− 30	46
	R	Occipital Pole	0.043	28	5.18	4.52	< 0.001	28	− 94	− 12
	L	Angular Gyrus	< 0.001	72	5.06	4.43	< 0.001	− 54	− 57	33
	R	Superior Temporal Gyrus, posterior division	0.003	42	4.94	4.35	< 0.001	63	− 24	− 5
	L	Precentral Gyrus	< 0.001	155	4.92	4.33	< 0.001	− 49	8	33
	R	Angular Gyrus	0.002	44	4.64	4.13	< 0.001	39	− 55	43
	L	Inferior Frontal Gyrus, pars opercularis	0.004	40	3.96	3.63	< 0.001	− 52	20	15
Incongruent > Congruent	L	Precentral Gyrus	< 0.001	2902	11.6	7.68	< 0.001	− 34	− 23	46
	L	Occipital Fusiform Gyrus	< 0.001	796	7.2	5.76	< 0.001	− 28	− 79	− 14
	L	Precentral Gyrus	< 0.001	313	7.18	5.74	< 0.001	− 56	9	23
	R	Lateral Occipital Cortex, inferior division	< 0.001	732	7.14	5.72	< 0.001	38	− 86	− 9
	R	Precentral Gyrus	< 0.001	122	6.67	5.46	< 0.001	55	11	32
	L	Central Opercular Cortex	< 0.001	78	6.46	5.33	< 0.001	− 49	− 21	21
	L	Lateral Occipital Cortex, superior division	< 0.001	729	6.27	5.22	< 0.001	− 28	− 67	31
	R	Cingulate Gyrus, anterior division	< 0.001	199	6.23	5.19	< 0.001	4	27	16
	R	Superior Frontal Gyrus	< 0.001	410	6.14	5.14	< 0.001	24	− 8	58
	L	Frontal Orbital Cortex	< 0.001	104	6.08	5.1	< 0.001	− 28	18	− 16
	R	Insular Cortex	< 0.001	120	5.76	4.9	< 0.001	40	14	− 7
	R	Frontal Orbital Cortex	< 0.001	79	5.62	4.81	< 0.001	26	15	− 18
	R	Juxtapositional Lobule Cortex	< 0.001	247	5.57	4.78	< 0.001	8	3	46
	L	Insular Cortex	< 0.001	54	5.48	4.72	< 0.001	− 37	9	− 4
	R	Supramarginal Gyrus, posterior division	< 0.001	494	5.47	4.71	< 0.001	37	− 37	42
	L	Paracingulate Gyrus	0.007	36	5.31	4.61	< 0.001	− 12	46	2
	R	Lateral Occipital Cortex, superior division	< 0.001	134	5.28	4.58	< 0.001	30	− 74	23
	R	Precuneous Cortex	0.004	39	5.07	4.44	< 0.001	4	− 66	46
	L	Intracalcarine Cortex	< 0.001	163	4.92	4.34	< 0.001	− 12	− 77	10
	L	Cingulate Gyrus, anterior division	< 0.001	207	4.85	4.28	< 0.001	− 2	31	19
	R	Intracalcarine Cortex	< 0.001	147	4.76	4.23	< 0.001	7	− 73	10
	L	Cingulate Gyrus, posterior division	0.016	32	4.76	4.22	< 0.001	− 2	− 24	43
	L	Precuneous Cortex	0.011	34	4.72	4.19	< 0.001	− 4	− 57	32
	L	Cingulate Gyrus, posterior division	< 0.001	54	4.72	4.19	< 0.001	− 2	− 50	21
	R	Lateral Occipital Cortex, superior division	< 0.001	190	4.68	4.16	< 0.001	29	− 62	53
	R	Angular Gyrus	0.036	28	4.67	4.16	< 0.001	41	− 57	17
	L	Insular Cortex	0.001	44	4.26	3.85	< 0.001	− 35	− 4	16
	R	Superior Parietal Lobule	< 0.001	76	4.09	3.72	< 0.001	28	− 48	50
**Stroop (Incongruent > Neutral)**										
Symmetric > Asymmetric	L	Superior Frontal Gyrus	0.013	33	5.01	4.36	< 0.001	− 4	46	46
	R	Paracingulate Gyrus	0.002	43	4.84	4.24	< 0.001	5	35	30

Group differences between individuals with symmetric and asymmetric ACC sulcation patterns are also reported. Significance threshold is set at vertex-p-uncorrected < 0.001 and cluster-p-FWE-corrected < 0.05. Only one local maximum per significant cluster is listed.

*R* Right hemisphere, *L* Left hemisphere.

### Brain-behavior interactions

Neuroimaging analyses showed a significant difference in brain activity between individuals with symmetric and asymmetric aMCC sulcal patterns in the incongruent > neutral contrast of the Numerical Stroop task. A brain-behavior correlation analysis was performed to explore the relationship between brain activity and RTs associated with this effect. For each participant, the mean BOLD signal was extracted from the significant clusters resulting from the incongruent > neutral contrast in the Numerical Stroop task. Mean functional activity was then correlated with the differences in RTs between incongruent and neutral trials in the 4^th^ and 5^th^ quantiles (corresponding to the slowest responses) separately for individuals with symmetric and asymmetric PCS profiles (see^71^). No significant correlation was found.

## Discussion

The present study aimed at investigating the neurofunctional impact of individual variability of the aMCC sulcal pattern during tasks assessing response inhibition. Each participant performed the ANT and the Numerical Stroop tasks, and surface-based fMRI analyses were adopted to identify group differences in functional activity within the cingulate and the prefrontal cortex. Behavioral measures were collected to further explore group performance differences. In the following, we start by reviewing the results associated with the two tasks separately, and conclude by discussing the general implications of our findings.

### ANT

Functional activation associated with the incongruent > congruent and incongruent > neutral contrasts revealed a significant brain activation of the frontal cortex, including the paracingulate gyrus, as well as temporo-occipital regions. This pattern of brain activation is coherent with the original results reported by Fan and colleagues (2005) for the same task. The involvement of regions dorsally contiguous with the aMCC (i.e., the paracingulate gyrus and the pre-supplementary motor area) has been associated with response inhibition within both the Flanker and Stroop tasks^12^, suggesting increased neural recruitment to deal with conflicting information when compared with congruent and neutral trials. The larger brain activity change found for the incongruent > neutral contrast compared to the incongruent > congruent contrast partly mirrors the differences in task difficulty as revealed by the mean RTs (incongruent > congruent > neutral).

When investigating differences in brain activity and behavioral measures depending on aMCC sulcation patterns, no significant result was found, neither in brain activity nor in behavioral measures. Hence, we failed to replicate the results by Cachia et al.^15^ and Del Maschio et al.^16^, who reported behavioral advantages related to asymmetric aMCC sulcal pattern associated with the incongruency effect in the same task. A possible reason for non-replication is related to the overall study design and differences in the group classification between studies. Cachia et al.^15^ classified their sample based on a two-level index (i.e., leftward—but not rightward—asymmetry, and symmetry), which was different from our classification, whereas Del Maschio et al.^16^ adopted a more comprehensive four-level index, with leftward asymmetry being the most common pattern. Additionally, the study had a complex cross-sectional design and included subgroups based on language experience. Sample inhomogeneity may have led to a leftward asymmetry overrepresentation in both studies.

The analysis of RTs divided into quantiles revealed a significant 3-way interaction between aMCC sulcal pattern, Condition, and Quantile. Considering the delta plots, individuals with asymmetric patterns showed positive linear slopes for both incongruent > congruent and incongruent > neutral effects. On the other hand, these effects changed nonlinearly as a function of time for individuals with symmetric patterns and, after an initial increase, the slope became flattened (and even negative) for the slowest responses. The leveling-off of a positive slope and negative-going components of delta plots have been typically associated with response inhibition in the Flanker task when using manipulations of the arrow direction as experimental conditions^60–62^. Ridderinkhof and colleagues^58,61^ suggested that in tasks requiring inhibitory control, incongruent stimuli prompt rapid and automatic activation of inappropriate responses, leading to large interference effects when responses are fast. Following an initial growth, this interference would be actively inhibited over time, and its influence on RTs reduced, leading to negative slopes^57,58^. The top-down mechanism responsible for the selective suppression of incorrect responses would require time to build up; hence changes in the slope direction would mainly affect the slowest quantiles, being proportional to the efficiency of inhibitory control^70^. We suggest that participants with a symmetric sulcal pattern were more efficient in suppressing incongruent responses in the slowest quantiles, leading to a pronounced leveling-off and to a negative change of the slope for this group. This finding suggests an advantage in response inhibition associated with a symmetric aMCC sulcal pattern. Without a significant difference in brain functional activity between groups, this advantage may be interpreted by focusing on the relationship between morphological symmetry and the structural organization of the brain. Symmetric brains show greater transcallosal structural connectivity than asymmetric ones, entailing a faster inter-hemispheric information transfer^72–74^. Moreover, the Flanker task is associated with bilateral information processing, as shown by evidence of symmetric task-related brain functional activity and connectivity in prefrontal clusters^75^. We suggest that, for individuals with symmetric sulcal patterns, the greater transcallosal structural connectivity may have promoted better transfer and integration of bilateral information. As a result, a more symmetric neuroanatomical organization would be associated with greater inhibitory control efficiency during the task. This interpretation is partially in contrast with the findings of Del Maschio et al.^16^ and Cachia et al.^15^. However, as previously mentioned, there are fundamental differences in the sample classification implemented by the two studies and ours. Moreover, the adoption of delta plot analyses may also have glimpsed aspects of the temporal dynamics of inhibitory control associated with differences in information transfer that could have been gone unnoticed in the previous experiments. As the functional measures in our study do not allow us to test adequately for changes in inter-hemispheric connectivity, this hypothesis should be tested by future studies. We also suggest that future research on this topic would benefit from larger samples (in the order of the hundred/s of participants) which would allow researchers to adopt more detailed classification systems (e.g., 4-levels classification, see^16^).

### Numerical stroop

Irrespective of aMCC sulcal pattern, the incongruent > congruent contrast activated a large set of frontal, insular, parietal, and occipital regions, including the left paracingulate gyrus and the bilateral ACC. The activation of the ACC has been frequently reported for the Stroop effect, both using the original color-word version^11,12,76^ and in versions adopting numbers as experimental stimuli^36,77–80^. The incongruent > neutral contrast was associated with the activation of frontal, parietal, and occipital areas, but no significant cluster was found in medial regions of the frontal cortex. The larger brain activity changes found for the incongruent > congruent contrast as compared to the incongruent > neutral contrast may reflect differences in task difficulty, as revealed by the mean RTs incongruent > neutral > congruent.

When investigating differences in brain activity depending on aMCC sulcal pattern, a significant difference was found for the incongruent > neutral contrast. Compared with individuals with a symmetric distribution of the PCS, individuals with an asymmetric aMCC sulcal pattern showed greater activation of the bilateral medial wall of the frontal lobe. In particular, two main clusters were reported: one located on the left medial frontal gyrus and a second located on the right paracingulate gyrus.. Compared with a neutral baseline, incongruency lead to greater activation of the paracingulate gyrus in individuals with symmetric patterns. Functional activity in this region has been associated with task difficulty^42,43,81^. Therefore, one might speculate that participants with a symmetric PCS distribution may have experienced greater effort in inhibiting automatic incongruent responses, leading to increased aMCC activity. However, here we simply emphasize that the same task resulted in a functional difference located on the PCS between individuals with symmetric and asymmetric sulcal patterns. This effect, in the opposite direction with respect to the Flanker task, can also be attributed to brain asymmetries and hemispheric specialization. Inhibitory control during the Stroop Task involves partially lateralized processing^12,75,82,83^. Moreover, information transfer is more efficient between spatially contiguous areas within the same hemisphere rather than between contralateral regions through callosal fibers^72–74^. According to Cachia and colleagues^14^, individuals with an asymmetric aMCC sulcal pattern would exhibit a more efficient inhibitory control because hemispheric specialization would prompt fast lateralized intra-hemispheric information transfer. On the other hand, individuals with morphological symmetries would rely on slower inter-hemispheric transfer to process information relevant to inhibit automatic responses. Based on our results, we suggest that the clusters of increased functional activity of bilateral aMCC in individuals with a symmetric aMCC sulcal pattern reflect the difficulty of integrating information arising from the two conjointly activated hemispheres. The process of updating and combining information would arguably be costly in terms of response time. The increased cost associated with updating and combining information would represent a possible explanation behind the recurrently reported asymmetric advantage in dealing with incongruent information during the Stroop task^13,14,17,18^. The contribution of individual cingulate morphology in determining significant functional differences was limited to the incongruent > neutral contrast. We suggest that this effect may be limited to interference (incongruent > neutral) rather than incongruency (incongruent > congruent) effects, that tackle distinct aspects of this specific version of the Stroop task. Therefore, this difference would be glimpsed only when contrasting incongruency with a neutral “intermediate” baseline rather than a “facilitating” congruent condition. This finding points towards a modest, yet measurable, morphology-related functional difference between the two groups when processing task interference.

The analysis of RTs divided into quantiles revealed a significant 3-way interaction between aMCC sulcal pattern, Condition, and Quantile. Considering the delta plots, when compared over the incongruent > neutral effect, participants with symmetric patterns showed a positive slope, suggesting an increasing interference effect as a function of the quantile. Participants with asymmetric patterns showed an initial increase followed by a flattening of the slope. Remember that slower trials manifest the most the effect of selective response suppression, which is typically represented by a leveling-off of the delta-plot slope^70,84^. We suggest that for individuals with asymmetric aMCC sulcal pattern after an initial increase of the interference effect, delta plot components in slow quantiles reflect efficient inhibition of incorrect automatic responses. In contrast, the almost linear increase of the incongruent > neutral effect for individuals with symmetric aMCC sulcal pattern may represent the need to combine responses from bilaterally activated cortices. Therefore, this increasingly pronounced interference effect as time passes would be caused by the difficulty associated with the integration process.

### Concluding remarks

This study provides the first evidence of the neurofunctional signature behind the modulation of inhibitory control in individuals with a variable sulcal morphology of the aMCC. While both the ANT and the Numerical Stroop tasks were associated with increased aMCC activity and longer RTs for incongruency trials, only the latter showed neurofunctional results supporting the notion of advantages associated with asymmetric aMCC sulcal patterns. Delta plots of behavioral effects revealed a symmetric-related advantage for the ANT and an asymmetric-related advantage for the Numerical Stroop task. Despite the two tasks being similar, previous imaging studies have shown larger functional asymmetries during the Stroop task compared with the Flanker, which was more symmetric overall^75^. The aMCC sulcal patterns might thus interact with the degree of functional symmetry/asymmetry of executive tasks, resulting in either beneficial or detrimental effects at the behavioral level. Based on our functional results, the significant group difference found only in the Numerical Stroop task implies that advantages would be more pronounced in the case of individuals with an asymmetric PCS while performing functionally asymmetric tasks, thanks to fast intra-hemispheric transfer. This phenomenon, previously investigated only at the morphological level, defines a brain structural and functional relationship that is arguably determined during early development and still impacts cognitive abilities in young adults decades later. Such interaction expands the still marginal knowledge on the neurofunctional impact of cortical gyrification patterns and paves the way for future research investigating other cognitive processes subserved by the ACC and aMCC, such as decision making or language control (see^85,86^).

## Supplementary Information

Supplementary Information.

## Data Availability

The datasets generated and analyzed during the current study are not publicly available to preserve participant’s privacy, but are available from the corresponding author on reasonable request.
