# Supplementary material for: Cingulate cortex morphology impacts on neurofunctional activity and behavioral performance in interference tasks
Source: Sci Rep. 2022 Aug 11;12:13684. doi: 10.1038/s41598-022-17557-6 (PMC9372177; doi:10.1038/s41598-022-17557-6)

Supplementary Materials

| **ANT** |  |  |  |  |  |  |  |  |  |  |
| --- | --- | --- | --- | --- | --- | --- | --- | --- | --- | --- |
| **Contrast** | **Hemisphere** | **Region (Harvard-Oxford)** | **cluster p(FWE-corr)** | **k (mm3)** | **T value** | **Z score** | **peak p(unc)** | **x** | **y** | **z** |
| Congruent > Neutral | R | Occipital Fusiform Gyrus | <0.001 | 5077 | 14.11 | 65535.00 | <0.001 | 20 | -82 | -12 |
|  | L | Temporal Pole | <0.001 | 375 | 5.30 | 4.60 | <0.001 | -42 | 16 | -18 |
|  | R | Superior Frontal Gyrus | <0.001 | 409 | 4.82 | 4.26 | <0.001 | 22 | 0 | 56 |
|  | L | Superior Frontal Gyrus | 0.05 | 200 | 4.14 | 3.76 | <0.001 | -24 | -6 | 48 |
|  |  |  |  |  |  |  |  |  |  |  |
|  |  |  |  |  |  |  |  |  |  |  |
| Incongruent > Neutral (Peak FWE p<0.05 corrected) | L | Occipital Pole | <0.001 | 2164 | 15.03 | 65535.00 | <0.001 | -16 | -92 | -10 |
|  | R | Occipital Pole | <0.001 | 3779 | 13.41 | 65535.00 | <0.001 | 20 | -90 | -6 |
|  | L | Lateral Occipital Cortex | <0.001 | 948 | 7.95 | 6.15 | <0.001 | -30 | -58 | 62 |
|  | L | Precentral Gyrus | <0.001 | 550 | 7.44 | 5.88 | <0.001 | -24 | -10 | 46 |
|  | R | Middle Frontal Gyrus | <0.001 | 478 | 7.41 | 5.87 | <0.001 | 32 | -2 | 50 |
|  | R | Frontal Orbital Cortex | <0.001 | 248 | 7.03 | 5.66 | <0.001 | 36 | 22 | -20 |
|  | R | Cerebellum | <0.001 | 135 | 6.69 | 5.47 | <0.001 | 4 | -70 | -34 |
|  | R | Paracingulate Gyrus | <0.001 | 175 | 6.43 | 5.32 | <0.001 | 4 | 8 | 50 |
|  | L | Cerebellum | <0.001 | 17 | 6.19 | 5.17 | <0.001 | 0 | -58 | -30 |
|  | L | Precentral Gyrus | <0.001 | 68 | 6.15 | 5.15 | <0.001 | -48 | 4 | 34 |
|  | L | Frontal Orbital Cortex | 0.001 | 38 | 5.78 | 4.92 | <0.001 | -36 | 24 | -2 |
|  | R | Lingual Gyrus | 0.009 | 11 | 5.74 | 4.89 | <0.001 | 2 | -82 | -4 |
|  | R | Cerebellum | 0.011 | 9 | 5.64 | 4.82 | <0.001 | 6 | -72 | -22 |
|  | R | Paracingulate Gyrus | 0.035 | 1 | 5.43 | 4.69 | <0.001 | 8 | 30 | 32 |
|  | R | Precentral Gyrus | 0.029 | 2 | 5.42 | 4.68 | <0.001 | 48 | 6 | 32 |
|  |  |  |  |  |  |  |  |  |  |  |
| Incongruent > Congruent | R | Inferior Temporal Gyrus | <0.001 | 4882 | 9.75 | 6.98 | <0.001 | 44 | -60 | -10 |
|  | L | Lateral Occipital Cortex | <0.001 | 4178 | 8.08 | 6.21 | <0.001 | -38 | -86 | 8 |
|  | L | Precentral Gyrus | <0.001 | 675 | 5.75 | 4.90 | <0.001 | -34 | -4 | 52 |
|  | R | Middle Frontal Gyrus | <0.001 | 967 | 4.99 | 4.38 | <0.001 | 30 | -4 | 60 |
|  |  |  |  |  |  |  |  |  |  |  |
| Alerting Effect | -- | -- | -- | -- | -- | -- | -- | -- | -- | -- |
|  |  |  |  |  |  |  |  |  |  |  |
| Orienting Effect | -- | -- | -- | -- | -- | -- | -- | -- | -- | -- |

Table s1. t-contrast results for the effects detected in the ANT task (volume-based analysis). Significance threshold is set at voxel p-uncorrected < 0.001 and cluster-p-FWE-corrected < 0.05. Only one local maximum per significant cluster is listed. R = Right hemisphere. L = Left hemisphere. The Incongruent > Neutral contrast is reported at voxel p-FWE corrected < 0.05 threshold, since the voxel p-uncorrected < 0.001 threshold lead to large but uninterpretable clusters of functional activity.

| **Stroop** |  |  |  |  |  |  |  |  |  |  |
| --- | --- | --- | --- | --- | --- | --- | --- | --- | --- | --- |
| **Contrast** | **Hemisphere** | **Region (Harvard-Oxford)** | **cluster p(FWE-corr)** | **k (mm3)** | **T value** | **Z score** | **peak p(unc)** | **x** | **y** | **z** |
| Congruent > Neutral | L | Caudate | 0.03 | 223 | 5.26 | 4.57 | <0.001 | -4 | 4 | 20 |
|  |  |  |  |  |  |  |  |  |  |  |
| Incongruent > Neutral | L | Supramarginal Gyrus | <0.001 | 1940 | 5.81 | 4.94 | <0.001 | -58 | -52 | 28 |
|  | L | Middle Frontal Gyrus | <0.001 | 1515 | 5.37 | 4.65 | <0.001 | -42 | 10 | 34 |
|  | L | Paracingulate Gyrus | <0.001 | 721 | 4.86 | 4.29 | <0.001 | -6 | 44 | 24 |
|  | R | Frontal Operculum Cortex | 0.002 | 434 | 4.85 | 4.29 | <0.001 | 46 | 20 | -2 |
|  | R | Angular Gyrus | 0.010 | 282 | 4.83 | 4.27 | <0.001 | 60 | -50 | 32 |
|  | L | Superior Frontal Gyrus | 0.017 | 235 | 4.10 | 3.73 | <0.001 | -4 | 22 | 54 |
|  |  |  |  |  |  |  |  |  |  |  |
| Incongruent > Congruent (Peak FWE p<0.05 corrected) | L | Postcentral Gyrus | <0.001 | 3389 | 10.34 | 7.21 | <0.001 | -42 | -26 | 56 |
|  | R | Occipital Pole | <0.001 | 366 | 8.78 | 6.55 | <0.001 | 24 | -94 | -10 |
|  | L | Supplementary Motor Cortex | <0.001 | 520 | 7.63 | 5.98 | <0.001 | -6 | -4 | 50 |
|  | R | Cerebellum | <0.001 | 490 | 7.56 | 5.95 | <0.001 | 26 | -50 | -28 |
|  | R | Precentral Gyrus | <0.001 | 342 | 7.35 | 5.84 | <0.001 | 26 | -12 | 56 |
|  | L | Lateral Occipital Cortex | <0.001 | 539 | 7.10 | 5.70 | <0.001 | -34 | -84 | -12 |
|  | R | Cingulate Gyrus | <0.001 | 145 | 6.86 | 5.57 | <0.001 | 8 | 30 | 20 |
|  | L | Frontal Orbital Cortex | <0.001 | 55 | 6.69 | 5.47 | <0.001 | -30 | 22 | -16 |
|  | L | Caudate | <0.001 | 170 | 6.64 | 5.44 | <0.001 | -12 | 8 | 6 |
|  | R | Thalamus | <0.001 | 106 | 6.50 | 5.36 | <0.001 | 12 | -20 | 8 |
|  | R | Lateral Occipital Cortex | <0.001 | 48 | 6.23 | 5.20 | <0.001 | 26 | -66 | 32 |
|  | R | Insular Cortex | <0.001 | 73 | 6.16 | 5.15 | <0.001 | 36 | 18 | -2 |
|  | R | Precentral Gyrus | 0.005 | 15 | 6.12 | 5.13 | <0.001 | 56 | 6 | 32 |
|  | R | Temporal Occipital Fusiform Cortex | 0.003 | 23 | 5.96 | 5.03 | <0.001 | 26 | -58 | -12 |
|  | L | Temporal Occipital Fusiform Cortex | 0.007 | 13 | 5.91 | 5.00 | <0.001 | -32 | -44 | -24 |
|  | R | Cingulate Gyrus | 0.023 | 3 | 5.83 | 4.95 | <0.001 | 6 | -24 | 36 |
|  | L | Brainstem | 0.004 | 19 | 5.78 | 4.91 | <0.001 | -8 | -24 | -6 |
|  | L | Insular Cortex | 0.002 | 28 | 5.70 | 4.86 | <0.001 | -34 | 6 | -4 |
|  | R | Frontal Orbital Cortex | 0.013 | 7 | 5.69 | 4.86 | <0.001 | 30 | 20 | -16 |
|  | R | Supramarginal Gyrus | <0.001 | 65 | 5.68 | 4.85 | <0.001 | 46 | -28 | 40 |
|  | R | Lateral Occipital Cortex | 0.013 | 7 | 5.62 | 4.81 | <0.001 | 36 | -84 | 8 |
|  | R | Cingulate Gyrus | 0.017 | 5 | 5.54 | 4.76 | <0.001 | 10 | 16 | 30 |
|  | R | Thalamus | 0.020 | 4 | 5.52 | 4.75 | <0.001 | 8 | -10 | -6 |
|  | L | Temporal Occipital Fusiform Cortex | 0.028 | 2 | 5.51 | 4.74 | <0.001 | -36 | -58 | -16 |
|  | R | Paracingulate Gyrus | 0.028 | 2 | 5.50 | 4.73 | <0.001 | 4 | 22 | 36 |
|  | L | Cerebellum | 0.035 | 1 | 5.44 | 4.69 | <0.001 | -10 | -56 | -24 |
|  | R | Superior Frontal Gyrus | 0.028 | 2 | 5.44 | 4.69 | <0.001 | 22 | 6 | 62 |
|  | L | Cerebellum | 0.035 | 1 | 5.42 | 4.68 | <0.001 | -12 | -54 | -26 |
|  | L | Paracingulate Gyrus | 0.035 | 1 | 5.39 | 4.66 | <0.001 | -8 | 48 | 10 |
|  | R | Occipital Fusiform Gyrus | 0.020 | 4 | 5.39 | 4.66 | <0.001 | 38 | -66 | -12 |
|  | L | Intracalcarine Cortex | 0.035 | 1 | 5.39 | 4.66 | <0.001 | -12 | -68 | 12 |
|  | R | Inferior Frontal Gyrus | 0.035 | 1 | 5.37 | 4.65 | <0.001 | 56 | 12 | 16 |
|  | L | Central Opercular Cortex | 0.035 | 1 | 5.37 | 4.65 | <0.001 | -52 | -20 | 18 |
|  |  |  |  |  |  |  |  |  |  |  |
| Neutral > Congruent (Peak FWE p<0.05 corrected) | L | Lingual Gyrus | <0.001 | 12350 | 19.05 | Inf | <0.001 | -14 | -88 | -8 |
|  | L | Precentral Gyrus | <0.001 | 623 | 8.39 | 6.36 | <0.001 | -36 | -24 | 50 |
|  | R | Cingulate Gyrus, anterior division | <0.001 | 103 | 7.52 | 5.93 | <0.001 | 14 | 0 | 38 |
|  | L | Precentral Gyrus | <0.001 | 111 | 6.46 | 5.33 | 0.003 | -8 | -26 | 46 |
|  | L | Precentral Gyrus | 0.001 | 36 | 6.39 | 5.29 | 0.003 | -4 | -28 | -8 |
|  | R | Precentral Gyrus | 0.002 | 20 | 6.29 | 5.23 | 0.005 | 58 | 4 | 32 |
|  | L | Insular Cortex | 0.007 | 10 | 6.22 | 5.19 | 0.006 | -32 | -2 | 12 |
|  | R | Cerebellum | 0.001 | 37 | 6.04 | 5.08 | 0.009 | 4 | -70 | -34 |
|  | R | Precuneous Cortex | 0.005 | 13 | 5.89 | 4.98 | 0.014 | 24 | -56 | 8 |
|  | L | Precentral Gyrus | 0.005 | 13 | 5.79 | 4.92 | 0.019 | -22 | -10 | 60 |
|  | R | Precentral Gyrus | 0.026 | 2 | 5.72 | 4.88 | 0.022 | 36 | -14 | 66 |
|  | L | Superior Frontal Gyrus | 0.033 | 1 | 5.51 | 4.74 | 0.039 | -20 | -10 | 52 |
|  | L | Cerebellum | 0.033 | 1 | 5.49 | 4.72 | 0.042 | -14 | -62 | -42 |

Table s2. t-contrast results for the effects detected in the Numerical Stroop task (volume-based analysis). Significance threshold is set at voxel p-uncorrected < 0.001 and cluster-p-FWE-corrected < 0.05. Only one local maximum per significant cluster is listed. R = Right hemisphere. L = Left hemisphere. The Incongruent > Congruent and Neutral > Congruent contrasts are reported at voxel p-FWE corrected < 0.05 threshold, since the voxel p-uncorrected < 0.001 threshold lead to large but uninterpretable clusters of functional activity.

Figure S1. Attention Network Test. a. Examples of central, double, and spatial cues; b. examples of congruent, icongruent, and neutral stimuli; c. task procedure.


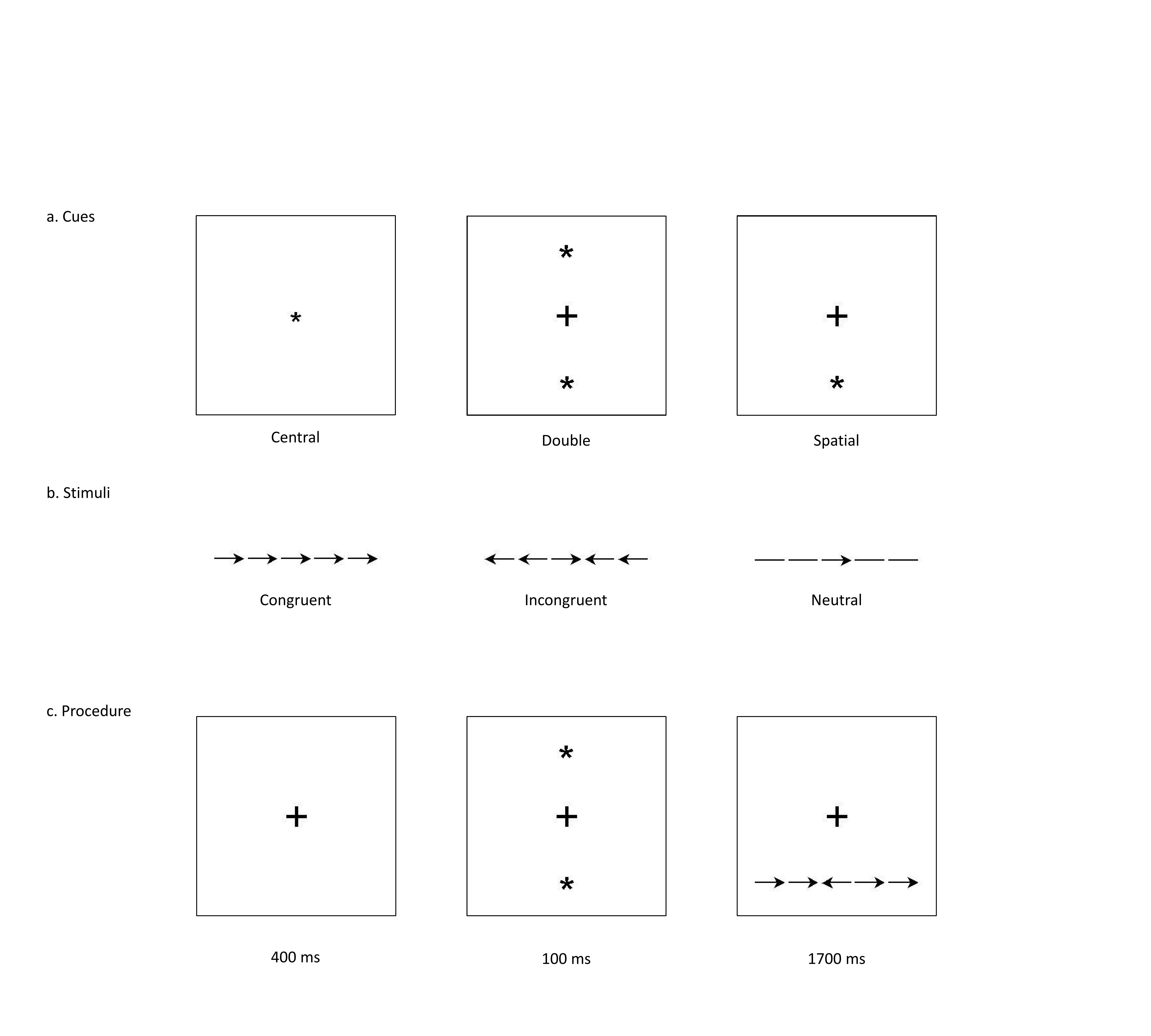


Figure S2. Plots of the RTs for each condition observed in the Attention Network Task and Numerical Stroop task, as a function of quantiles. Incong = Incongruent. Cong = Congruent. Neutr. = Neutral. PPs = Participants. PCS = Paracingulate Sulcus.


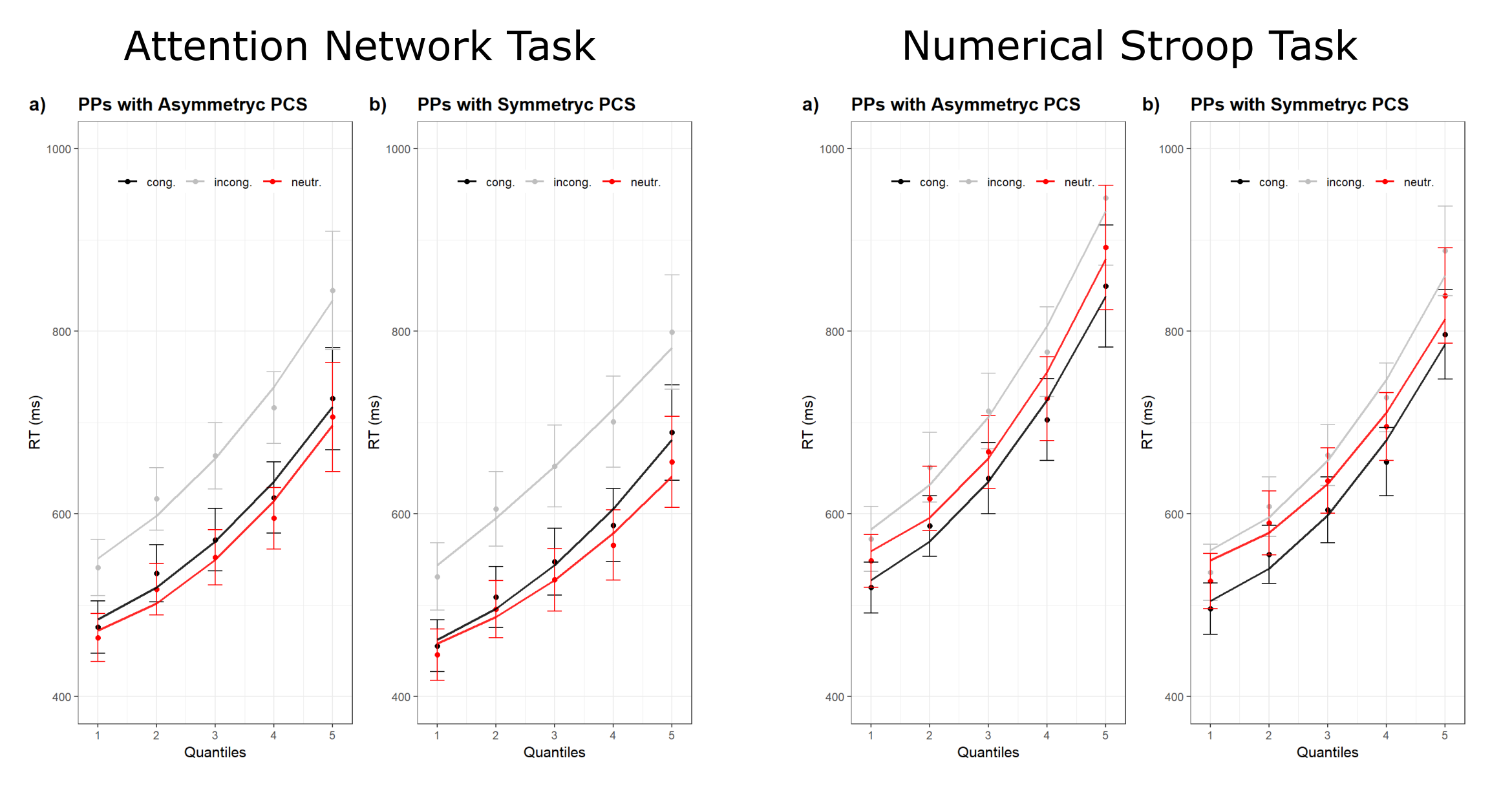


Figure S3. Incongruent>neural contrast plotted separately for individuals with Symmetric and Asymmetric aMCC sulcation.Significant results are shown at cluster level FWE-corrected for multiple comparisons p-value < 0.05, and vertex level uncorrected p-value < 0.001.


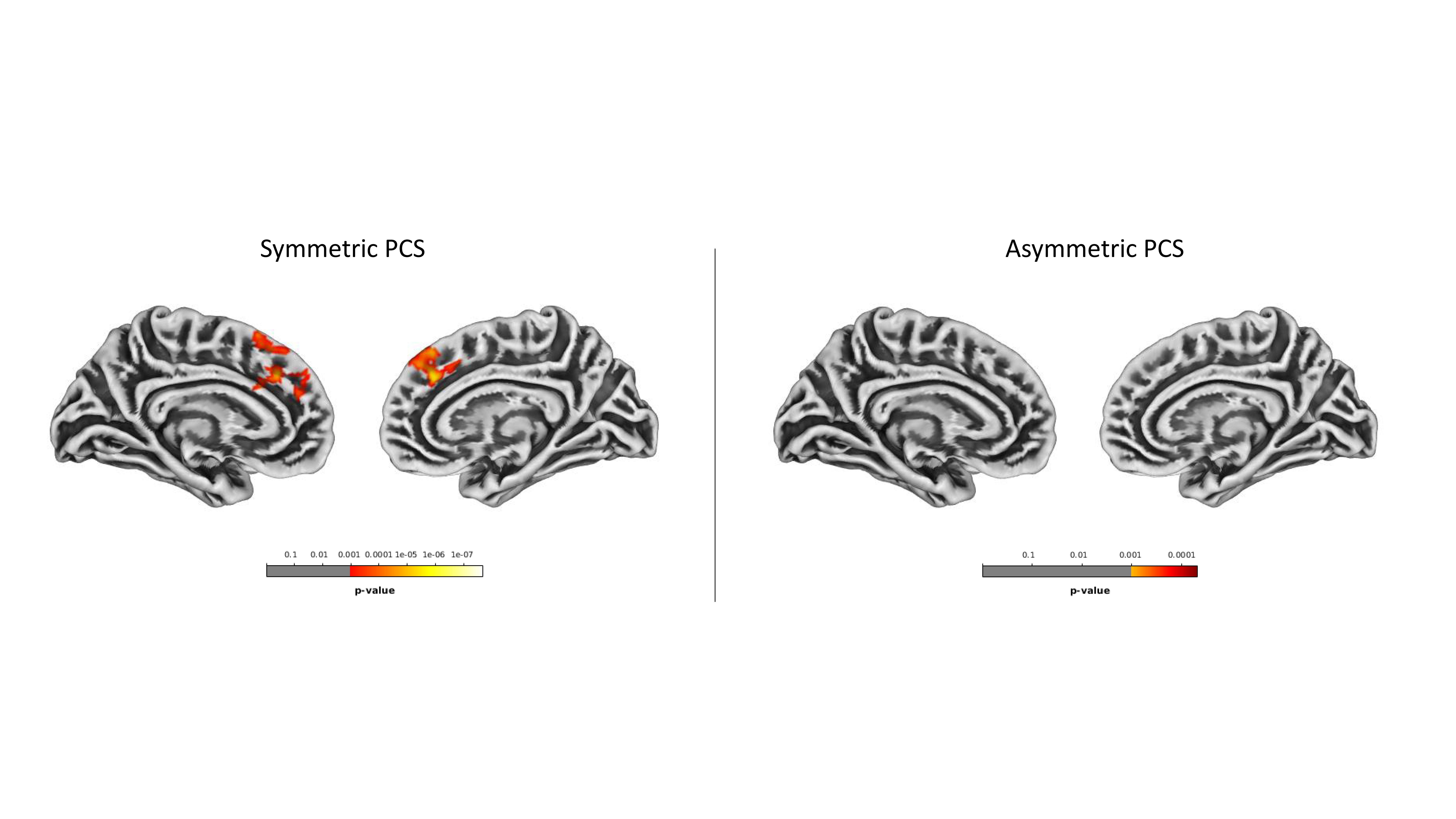

Supplement: Supplementary file 1 — Supplementary Information. [file 41598_2022_17557_MOESM1_ESM.doc]
